# Monensin, an Antibiotic Isolated from *Streptomyces Cinnamonensis*, Regulates Human Neuroblastoma Cell Proliferation via the PI3K/AKT Signaling Pathway and Acts Synergistically with Rapamycin

**DOI:** 10.3390/antibiotics12030546

**Published:** 2023-03-09

**Authors:** Sema Serter Kocoglu, Mücahit Secme, Ceren Oy, Gözde Korkusuz, Levent Elmas

**Affiliations:** 1 Department of Histology and Embryology, Faculty of Medicine, Balikesir University, Balikesir 10145, Turkey; 2 Department of Medical Biology, Faculty of Medicine, Ordu University, Ordu 52000, Turkey; 3 Department of Histology and Embryology, Faculty of Medicine, Bursa Uludag University, Bursa 16059, Turkey; 4 Department of Medical Biology, Faculty of Medicine, Bakırçay University, İzmir 35665, Turkey

**Keywords:** *Streptomyces cinnamonensis*, monensin, rapamycin, neuroblastoma, anticancer, PI3K/AKT pathway

## Abstract

Neuroblastoma is the most common extracranial childhood tumor and accounts for approximately 15% of pediatric cancer-related deaths. Further studies are needed to identify potential therapeutic targets for neuroblastoma. Monensin is an ionophore antibiotic obtained from *Streptomyces cinnamonensis* with known antibacterial and antiparasitic effects. No study has reported the effects of monensin on SH-SY5Y neuroblastoma cells by targeting the PI3K/AKT signaling pathway. The aim of this study was to investigate the antiproliferative effects of monensin alone and in combination with rapamycin in human SH-SY5Y neuroblastoma cells mediated by the PI3K/AKT signaling pathway. The effects of single and combination applications of monensin and rapamycin on SH-SY5Y cell proliferation were investigated by XTT, and their effects on the PI3K/AKT signaling pathway by RT-PCR, immunohistochemistry, immunofluorescence, and Western blotting. The combined effects of monensin and rapamycin on SH-SY5Y proliferation were most potent at 72 h (combination index < 1). The combination of monensin and rapamycin caused a significant decrease in the expression of P21RAS, AKT, and MAPK1 genes. Single and combined administrations of monensin and rapamycin caused a significant decrease in PI3K/AKT expression. Our results showed for the first time that monensin exerts an antiproliferative effect by targeting the PI3K/AKT signaling pathway in neuroblastoma cells. It is suggested that monensin and its combination with rapamycin may be an effective therapeutic candidate for treating neuroblastoma.

## 1. Introduction

Neuroblastomas originate from the neural crest and are the most common extracranial solid tumor in children. Each year, 700 cases are diagnosed in the United Nations alone, and the average age of diagnosis for those younger than five years is 17 months [1]. Neuroblastomas account for 15% of childhood cancer-related deaths, and approximately 40% of patients are younger than one year of age at diagnosis [2]. Tumors arise in tissues associated with the sympathetic nervous system, such as the adrenal medulla or paraspinal ganglion. They may present as mass lesions in the neck, chest, abdomen, and pelvis. The incidence of neuroblastoma is 10.2 cases per 1 million children under the age of 15 [3]. Depending on the patient’s age, structure, localization, and tumor stage, methods such as chemotherapy, radiotherapy, surgery, gene therapy, and immunotherapy are appropriate for treating pediatric neuroblastoma [4,5]. Despite the improvements in the disease treatment methods, the survival rate of below 40% is not satisfactory, and there is an urgent need for new therapeutic agents.

Monensin (mon) is a polyether ionophore antibiotic isolated from the bacterium *Streptomyces cinnamonensis* and widely used to treat fungal, bacterial, and parasitic infections [6,7]. The anticancer activity of mon in various cancers such as ovarian cancer [8], prostate [9], lung [10], pancreatic [11], and breast cancer [12] has been reported. Mon can strongly inhibit tumor endothelial cells and cancer stem cells [7]. It has been reported that monensin inhibits the proliferation of ovarian cancer cells, triggers apoptosis, and has a synergistic effect with oxaliplatin [13]. Studies show that monensin can inhibit many cancer-related pathways (E2F/DP1, STAT1/2, NFκB, AP-1, and Elk-1/SRF). Wang et al. reported that monensin exerts its proliferation suppressive effect by inhibiting multiple growth pathways, such as EGFR [14]. However, the anticancer mechanism of mon in SH-SY5Y neuroblastoma cells has not been reported yet.

It has been reported that overexpression of cell viability pathways is dominant in neuroblastoma initiation and progression stages. Abnormal activation of the PI3K/AKT survival pathway is vital for neuroblastoma [15]. AKT plays a vital role in biological processes such as cell proliferation, differentiation, apoptosis, and metabolism. There are three types of isoforms in mammals (AKT1, AKT2, and AKT3), and in most tissues, the predominant form is AKT1 [16]. In the literature, semiquantitative immunohistochemical studies have shown that active AKT (pAKT) expression is high in neuroblastoma and is associated with poor outcomes [17].

Moreover, several new drugs, such as geldanamycin and rapamycin (rap), directly alter the expression of AKT in tumoral cells [16]. The mammalian target of rapamycin (mTOR) is an essential regulator of cell development and metabolism. Rap is a specific prophylactic for mTOR that binds FKBP12 to form a molecular complex that inhibits mTOR activity and has antifungal, immunosuppressive, antiproliferative, and anti-aging effects [18,19,20]. Rap and its derivatives currently have anticancer effects against tumors such as lymphoma, renal cell carcinoma, prostate, breast, and lung [21]. Rapamycin is an essential regulator of the PI3 kinase signaling pathway [18]. Investigating the underlying mechanisms and how cancer cells resist PI3K/AKT pathway inhibition is of interest in anticancer studies. Furthermore, depicting the relationship between the PI3K/AKT pathway of mon alone and in combination with mon in tumor development may provide new treatment strategies to change the aggressive nature of NB.

This study investigated the anticarcinogenic effects of mon, alone or in combination with rap, targeting the PI3K/AKT signaling pathway in neuroblastoma cells. Overall, the results of the presented study reveal a previously unknown mechanism of mon against neuroblastoma.

## 2. Results

### 2.1. The Effect of Single and Combined Administration of Mon and Rap on SH-SY5Y Cell Viability

**Figure 1:**
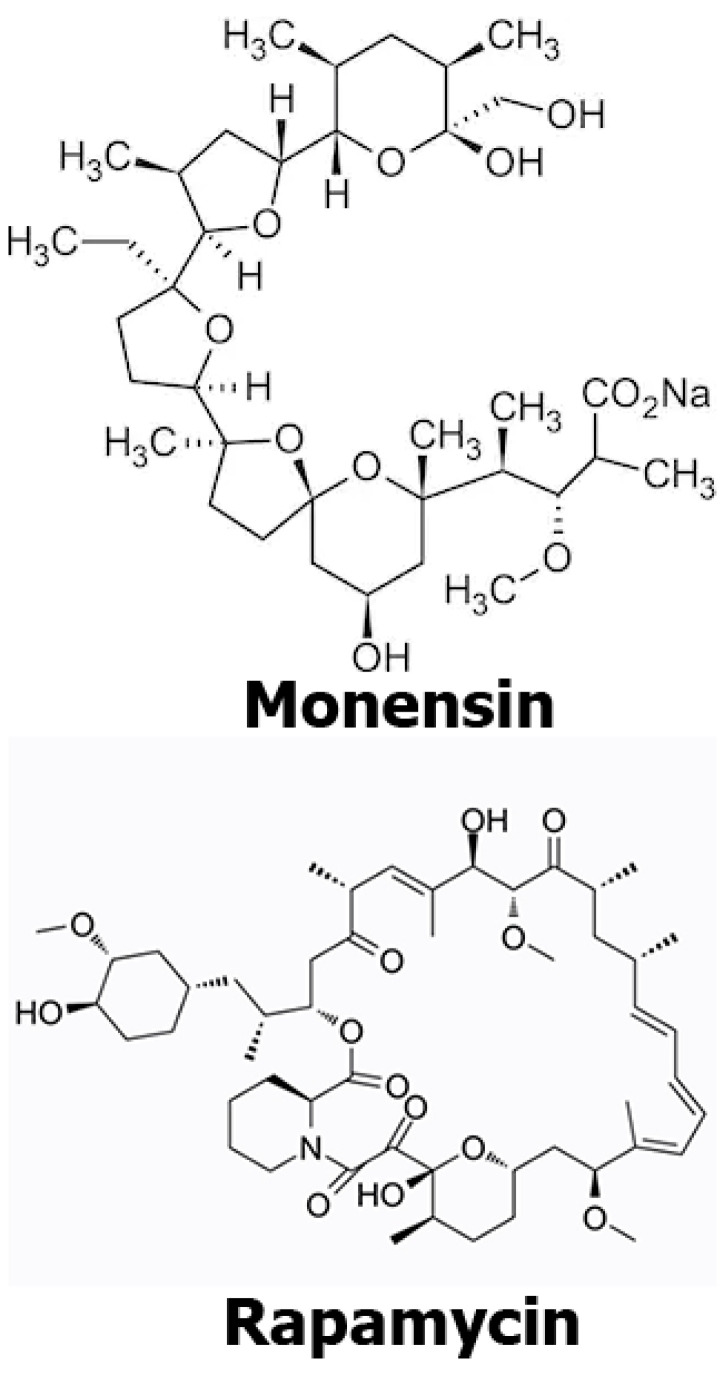
Chemical structures of mon and rap.

The combination effects of mon (Figure 1) and rap (Figure 1) on SH-SY5Y cells were analyzed using XTT assay. The combination of mon and rap at 72 h reduced SH-SY5Y cell viability more than their single application. In addition, the combined effects of mon and rap on SH-SY5Y cells were found to be most assertive at 72 h (CI < 1) (Table 1).

**Figure 2:**
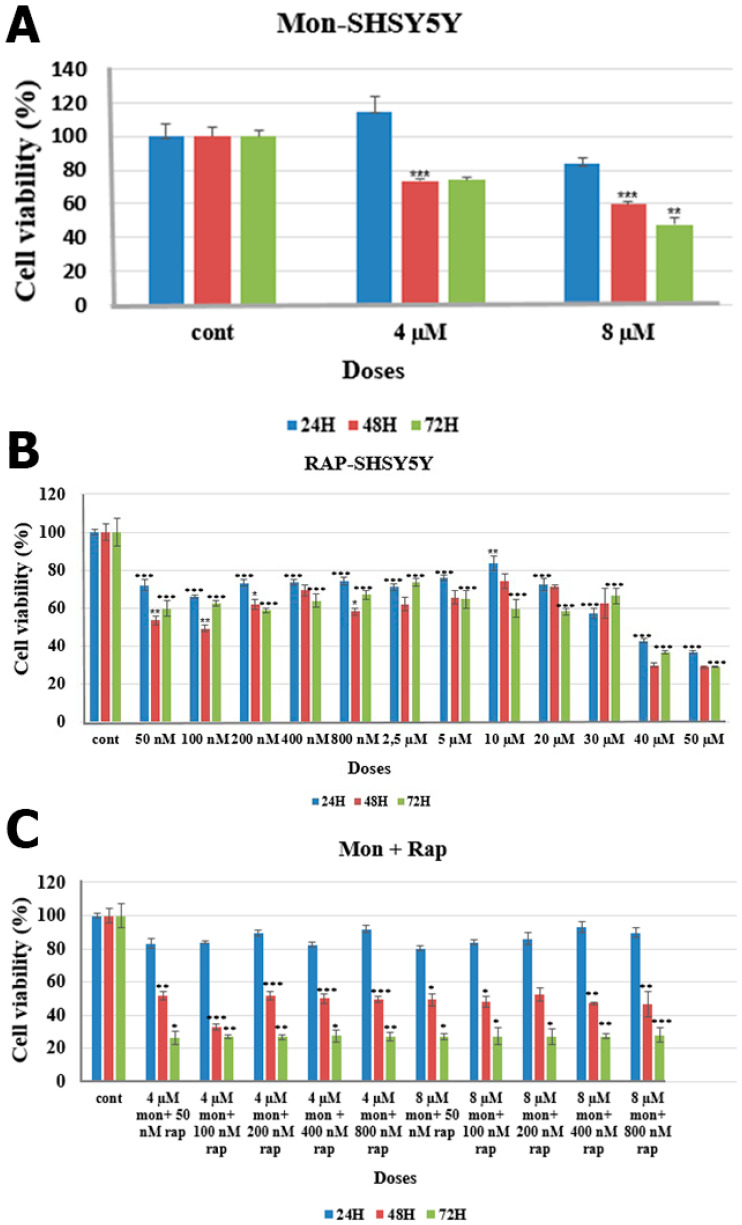
Effects of mon alone, rap alone, and combination of mon + rap on SH-SY5Y cell viability. (**A**) Effects of mon on cell viability. (**B**) Effects of rap on cell viability. (**C**) Effects of the combination of mon and rap on cell viability. Data are presented as mean ± SD. *n* = 3. *, **, and *** indicate statistical differences of *p* < 0.05, *p* < 0.01, and *p* < 0.001, respectively, compared to control group cells.

At 72 h, 4 µM and 8 µM mon were found to reduce SH-SY5Y cell viability to 74% and 47%, respectively (Figure 2A). In order to evaluate the effects of mon in combination with rap, the effects of rap alone on SH-SY5Y cell viability were also evaluated. Rap alone significantly reduced SH-SY5Y cell viability at 24, 48, and 72 h. Rap at 50 nM, 100 nM, 200 nM, 400 nM, and 800 nM at 72 h decreased cell viability to 59%, 62%, 58%, 63%, and 66%, respectively (*p* < 0.001) (Figure 2B). High efficacy of the combination of mon and rap was observed at 72 h. Combined applications of 4 µM mon with 50, 100, or 200 nM rap decreased SH-SY5Y cell viability to 26%, 27%, and 26%, respectively (*p* < 0.05) (Figure 2C).

The combination of mon and rap showed a substantial synergistic effect on SH-SY5Y cells at 72 h and low concentrations (CI < 1) (Table 1). For this reason, 72 h and the lowest concentration values were preferred in other experimental stages (50 nM rap + 4 µM mon).

### 2.2. Determination of the Effect of Single and Combined Doses of Mon and Rap on PI3K/AKT Protein Expression Using Immunohistochemistry

**Figure 3:**
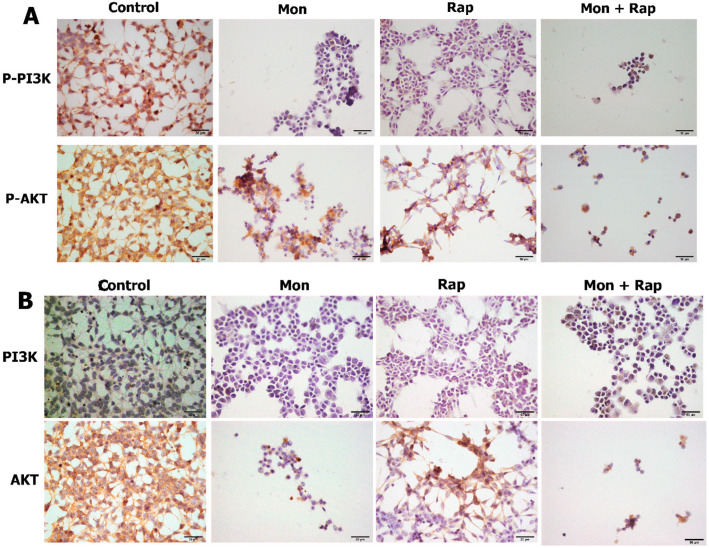
Immunohistochemical investigation of the effects of single and combined doses of mon and rap on PI3K and AKT expression in SH-SY5Y cells. (**A**) p-PI3K and p-AKT expression in different groups. (**B**) PI3K and AKT expression in different groups. p-AKT/AKT and p-PI3K/PI3K were expressed in the cytoplasm, cell membrane, and cytoplasmic extensions (brown) of SH-SY5Y cells. Bar: 50 µm.

**Figure 4:**
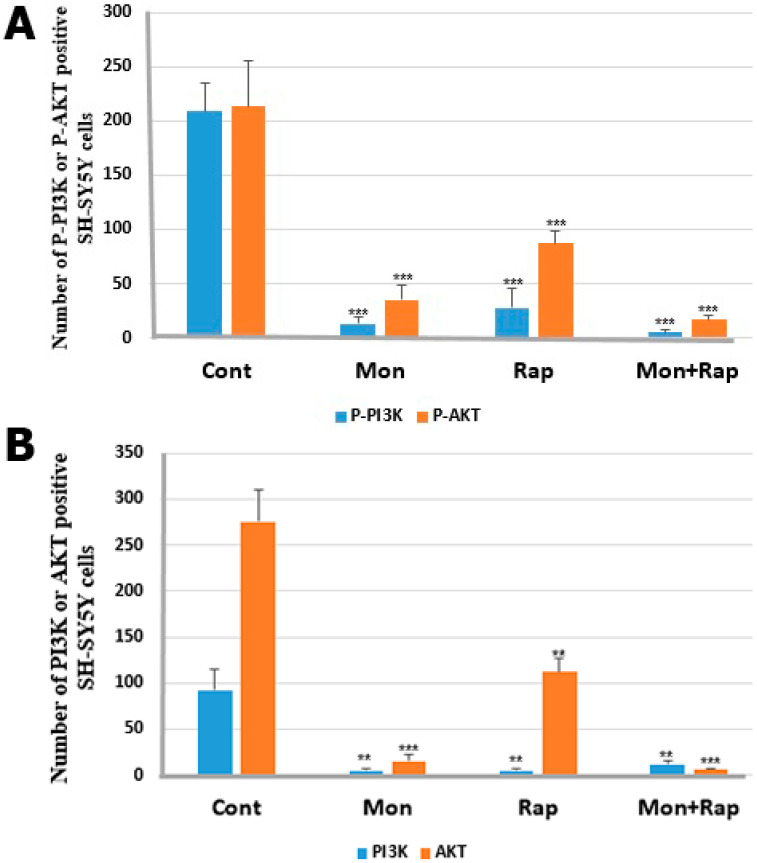
Statistical analysis of the effects of single and combined doses of mon and rap on the PI3K/AKT pathway. (**A**,**B**) Significant differences were found between control and treatment groups. Data are presented as mean ± SD. *n*=3. ** *p* < 0.01, *** *p* < 0.001.

Single and combined applications of mon and rap caused a decrease in the number of p-AKT/AKT- and p-PI3K/PI3K-positive SH-SY5Y cells. p-AKT/AKT and p-PI3K/PI3K were expressed in the cytoplasm, cell membrane, and cytoplasmic extensions of SH-SY5Y cells (Figure 3). Mon application caused the cells to change shape. It was observed that the cells gained a morphology close to round, and their extensions decreased. Although rap application alone did not change the morphology of the cell, when applied together with mon, the morphology of the cell then changed. The mean numbers of p-PI3K-positive SH-SY5Y cells were 209 ± 25 in the control group, 13 ± 6 (*p* = 0.000) in the mon group, 27 ± 18 (*p* = 0.000) in the rap group, and 5 ± 3 (*p* = 0.000) in the mon + rap group (Figure 4A). Meanwhile, the numbers of p-AKT-positive SH-SY5Y cells were 213 ± 42 in the control group and 35 ± 13 (*p* = 0.000), 87 ± 11 (*p* = 0.000), and 16 ± 5 (*p* = 0.000) in the mon, rap, and mon + rap groups, respectively (Figure 4A).

The mean number of PI3K-positive SH-SY5Y cells was 92.4±23 in the control group, while it was 4.8 ± 3 (*p* = 0.006) in the mon group, 5 ± 2 (*p* = 0.006) in the rap group, and 12 ± 4 (*p* = 0.008) in the mon + rap group (Figure 4B). Meanwhile, the number of AKT-positive SH-SY5Y cells was 275.6 ± 35 in the control group and 15.6 ± 7 (*p* = 0.000), 112 ± 15 (*p* = 0.001), and 6.6 ± 1 (*p* = 0.000) in the mon, rap, and mon + rap groups, respectively (Figure 4B).

### 2.3. Determination of the Effect of Single and Combined Doses of Mon and Rap on PI3K/AKT Protein Expression Using Immunofluorescence Method

**Figure 5:**
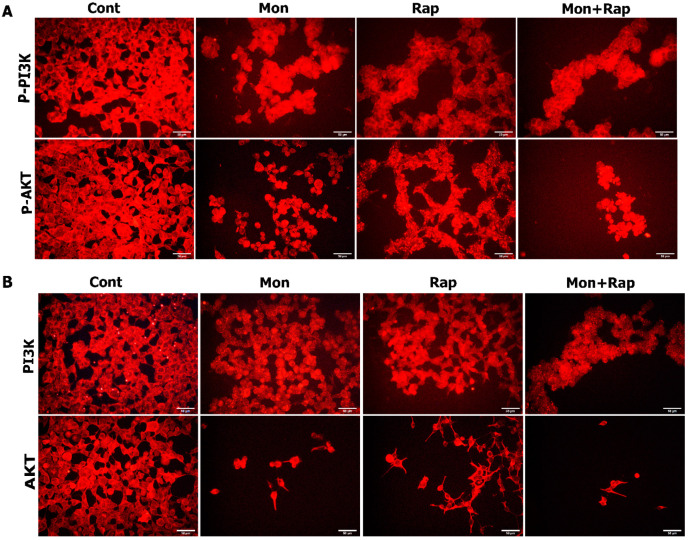
Evaluation of the effects of single and combined doses of mon and rap on the PI3K/AKT pathway using the immunofluorescence method. (**A**) Expressions of p-PI3K and p-AKT in different groups are labeled at the red fluorescent wavelength. (**B**) PI3K and AKT expressions in different groups are labeled at the red fluorescent wavelength. Bar: 50 µm.

Single and combined administrations of mon and rap caused a decrease in the expression of p-PI3K/PI3K- and p-AKT/AKT-positive SH-SY5Y cells (Figure 5). Expression of p-PI3K/PI3K and p-AKT/AKT was observed in SH-SY5Y cells in all groups (Figure 5). Expression of p-PI3K/PI3K and p-AKT/AKT was strong in the cytoplasm, membrane, and cytoplasmic extensions of the cell. The staining intensity of p-PI3K/PI3K and p-AKT/AKT was strong in the control group, whereas the staining intensity was decreased in the mon + rap combination (Figure 5).

### 2.4. Determination of the Effect of Single and Combined Doses of Mon and Rap on PI3K/AKT Protein Expression Using SDS-Polyacrylamide Gel Electrophoresis (SDS-PAGE) and Western Blotting

**Figure 6:**
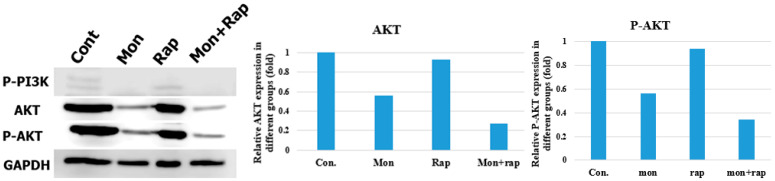
The effects of single and combined mon and rap on the PI3K/AKT pathway. Western blot assays were used to measure the expression of p-PI3K, AKT, and P-AKT in SH-SY5Y cells. Quantification of AKT and P-AKT proteins was performed and normalized to the GAPDH loading control.

Single and combined administrations of mon and rap resulted in decreased expression of the PI3K/AKT pathway. pPI3K and AKT/pAKT protein expression was decreased in mon, rap, and combination groups compared to the control group. In addition, densitometric analyses showed that AKT and p-AKT expression decreased in all groups compared to the control group (Figure 6).

### 2.5. Determination of the Effects of Single and Combined Doses of Mon and Rap on the Expression of Genes Associated with the PI3K/AKT Pathway Using RT-PCR Method

The combination of mon and rap resulted in decreased expression of genes associated with the PI3K/AKT pathway (Table 2). Expression of the p21RAS gene was significantly decreased in mon, rap, and combination groups. Meanwhile, AKT and MAPK1 gene expression was significantly decreased in the combination group, and no significant change was observed in the single mon and rap groups (Table 2). Although PIK3C3 and PTEN gene expression decreased 8- and 20-fold in the combination group, respectively, they were not found to be significant (Table 2).

## 3. Discussion

Neuroblastoma is the most common fetal extracranial solid tumor in childhood. It accounts for approximately 7–10% of pediatric malignancies [22]. Patients with neuroblastoma are divided into deficient, low, intermediate, and high-risk groups based on various characteristics. About half of the patients present with high-risk neuroblastoma; despite treatment protocols, survival in these patients is below 50% [22]. In conclusion, new biological or genetic-based therapies targeting the pathways responsible for malignant transformation and progression are required, especially for neuroblastoma, which is in the high-risk category.

Mon is a natural, fat-soluble antibiotic derived from *Streptomyces cinnamonensis* [6]. By providing Na+/H+ ion exchange in cell membranes, it disrupts the ion gradient and changes the cell physiology. It has antimicrobial, antiparasitic, and anticancer effects [6,14]. It has been reported that mon decreases renal cancer cell proliferation in a dose- and time-dependent manner [23]. In another study, it was shown that mon inhibited prostate cancer cell growth [9]. We have shown for the first time that mon decreases dose- and time-dependent SH-SY5Y cell proliferation.

The main signaling mechanisms important in cancer are the (PI3K)/AKT kinase chain, protein kinase C family (PKC), and mitogen-activated protein kinase (MAPK)/Ras. mTOR (mammalian target of rapamycin protein complex) is an essential kinase in the activation of the (PI3K)/AKT signaling pathway and has an active role in cell development, proliferation, angiogenesis, and protein synthesis. Because these pathways are disrupted by cancer, they are a crucial antitumor target. In clinical studies with mTOR inhibitors such as rap, a long-term tumor response has been achieved. Its effectiveness has been shown in many cancer types, including breast, lung, lymphoma, stomach, and sarcoma cancers. Therefore, mTOR inhibitors are an important target in many types of cancer [24,25,26]. Rap is a macrocyclic lactone isolated from *Streptomyces hygroscopicus.* It is widely used clinically as an antiproliferative drug and immunosuppressant. The number of studies on the effects of rap in tumor treatment is increasing. Lin et al. showed that 10, 20, 30, and 40 µM rap decreased SH-SY5Y cell proliferation in a dose- and time-dependent manner, and they preferred 20 µM rap for 24 h for treatment [27]. We have shown that rap at very low concentrations (50 nM) reduces SH-SY5Y cell proliferation in a dose- and time-dependent manner. Previous research revealed that rap inhibits neuroblastoma cell proliferation by reducing MYCN protein expression, which may be associated with the PI3K/AKT/mTOR pathway [28,29,30]. However, studies on the mechanism underlying the inhibitory effect of rap on neuroblastoma cell proliferation or the molecular pathways involved in this process have rarely been published. Our study investigated whether rap and mon could form an effective combination therapy in SH-SY5Y cells. Choi et al. showed that mon, when applied to lung cancer cells together with rap, exerted a strong synergistic effect and inhibited lung cancer cell growth [10]. Our study showed for the first time that mon with rap acts synergistically in SH-SY5Y cells and reduces the proliferation of SH-SY5Y cells.

The PI3K/AKT signaling pathway was investigated using immunohistochemistry, immunofluorescence, western blotting, and RT-PCR techniques to explore the mechanism by which mon and rap treatment reduced SH-SY5Y cell proliferation. Real-time PCR analyses showed significant changes in the mRNA levels of some genes associated with the PI3K/AKT pathway in mon-and-rap-treated SH-SY5Y cells. The combination of mon and rap caused decreased mRNA expression of AKT, MAPK1, and p21RAS genes in SH-SY5Y cells. In the next step, the effects of single and combined administrations of mon and rap on PI3K/AKT protein expression were investigated. Immunohistochemical results showed that single and combined administrations of mon and rap significantly reduced PI3K/pPI3K and AKT/pAKT expression compared to the control group. These results were also supported by immunofluorescence and Western blot results. Liao et al. reported that FoxM1 regulates the apoptosis and proliferation of human neuroblastoma cells via the PI3K/AKT pathway [31]. Zhang et al. showed that silencing of RRS1 reduced neuroblastoma cell proliferation via the PI3K/Akt/NF-kB pathway [15]. Our results showed for the first time that the combination of mon and rap in SH-SY5Y neuroblastoma cells reduces cell proliferation by targeting the PI3K/AKT pathway. PI3Ks constitute a family of lipid kinases that can phosphorylate the 3′-OH group on inositol phospholipids to form the second messenger phosphatidylinositol-3,4,5-trisphosphate (PI-3,4,5 P3). Class-I PI3Ks are heterodimers composed of a catalytic subunit (p110) and an adapter subunit (p85). RPTK (receptor protein tyrosine kinase) activation causes autophosphorylation of tyrosine residues. PI3K binds directly to phosphotyrosine residues of growth factor receptors via one or both SH2 domains in the adapter subunit (P85). This leads to allosteric activation of the catalytic subunit. This activation leads to the production of PIP3. The lipid product of PI3K, PIP3, binds to a subset of signaling proteins with membrane pleckstrin homology (PH) domains, such as PDK1 and Akt. Activated Akt modulates the function of multiple substrates involved in the regulation of cell survival, cell cycle progression, and cellular growth [32,33].

The phosphatidylinositol 3′-kinase (PI3K)/Akt pathway is one of the strongest signalings cascades that shows aberrant expression in various types of cancer. Recent studies show that pathological activation of Akt also frequently occurs in neuroblastoma and is associated with poor prognosis. Therefore, therapeutic targeting of PI3K/Akt/mTOR may offer a promising approach for the design of targeted molecular therapies in neuroblastoma [34]. Our results demonstrated that mon and rap exert their synergistic effect on SH-SY5Y cells using the PI3K/AKT signaling pathway at the protein level.

## 4. Materials and Methods

### 4.1. Cell Culture

The human neuroblastoma SH-SY5Y (ATCC^®^-: CRL-2266™) cell line was used in this study. Cells were incubated in DMEMF12 (Dulbecco’s Modified Eagle Medium) culture containing penicillin (20 units/mL), streptomycin (20 μg/mL), and 10% fetal bovine serum at 37 °C under 95% air and 5% CO_2_ pressure. Monensin sodium salt, Na^+^ ionohore (Figure 1) (Abcam, cat. no.: ab120499, Boston, MA, USA), and rap (Figure 1) (MedChemEkspress, cat. no.: HY-10219, Newark, NJ, USA) were dissolved in ethanol.

### 4.2. Cell Viability Assay

The effects of rap alone and in combination with mon on SH-SY5Y cell viability were analyzed using XTT (Biotium, Fremont, CA, USA). SH-SY5Y cells were seeded at 10^4^ cells/well in 96-well plates: 4 µM and 8 µM mon after 24 h; 50 nM, 100 nM, 200 nM, 400 nM, 800 nM, 2.5 µM, 5 µM, 10 µM, 20 µM, 30 µM, 40 µM, and 50 µM rap; 4 µM mon + 50 nM rap, 4 µM mon + 100 nM rap, 4 µM mon + 200 nM rap, 4 µM mon + 400 nM rap, 4 µM mon + 800 nM rap, 8 µM mon + 50 nM rap, 8 mon + 100 nM rap, 8 µM mon + 200 nM rap, 8 µM mon + 400 nM rap, and 8 µM mon + 800 nM rap combination doses. Each dose was administered three times: after 24, 48, and 72 h. The XTT method was applied according to the kit procedure of the manufacturer. Formazan formation was determined spectrophotometrically using a microplate reader (BioTek, Shoreline, WA, USA) at the reference range of 450 nm (reference wavelength 630 nm) [35]. Cell viability was calculated with the following formula:

Cell viability (%) = A of experimental well/A of control well × 100

The drug combination effects between mon and rap were analyzed using the Chou–Talalay method [11].

### 4.3. Immunohistochemistry

Cells were seeded on 8-well slides for immunohistochemical analysis. After 24 h, control, mon (4 µM), rap (50 nM), and mon + rap dose groups were formed and incubated for 72 h. The slides were washed with PBS and fixed with 4% paraformaldehyde. Then, they were treated with 3% H_2_O_2_/methanol for 10 min, washed with PBS, and blocked with blocking serum (Thermo Scientific, Waltham, MA, USA). They were next treated with primary antibodies at +4 °C for one night and washed with PBS. Following this, a specific secondary antibody was applied to the primary antibody. After washing with PBS, the secondary antibody was labeled with the streptavidin peroxidase enzyme complex. Proteins gained visibility with the DAB chromogen. The primary antibodies used and their dilutions were as follows: PI3K (1:2000, cat. no. BT-PHS00765, BTLab, Birmingham, UK), p-PI3K (1:2000, cat. no. BT-PHS00765, BTLab), AKT (1:2000, cat. no. BT-AP00334, BTLab), and p-AKT (1:2000, cat. no. BT-PHS00006, BTLab). In order to make the nuclei visible, they were counterstained with hematoxylin, washed in tap water until the dye ran, and covered with entellan after passing through increasing series of alcohol [36]. From each group, five different fields were photographed randomly at 200× magnification with light microscopy, and all SH-SY5Y cells showing AKT/p-AKT and PI3K/p-PI3K positivity were counted.

### 4.4. Immunofluorescence Staining

Cells were seeded on 8-well slides for immunohistochemical analysis. After 24 h, control, mon (4 µM), rap (50 nM), and mon + rap dose groups were formed and incubated for 72 h. The slides were washed with PBS and fixed with 4% paraformaldehyde. Then, they were treated with 3% H_2_O_2_/methanol for 10 min, washed with PBS, and blocked with blocking serum (Thermo Scientific, Waltham, MA, USA). They were next treated with primary antibodies at +4 °C for one night and washed with PBS. After that, they were washed with PBS and treated with Alexa fluor 555-goat anti-rabbit (1:200, cat. no. FNSA-0095). The samples were next covered with antifade mounting medium (Enzo Biochem Inc., Farmingdale, NY, USA) containing DAPI and left to dry overnight [36]. Examination of sections marked using the dual immunofluorescence method was carried out based on images taken with a digital camera (Olympus DP71 CCD color camera, 1.5 million pixels) using an Olympus BX–FLA Reflected Light Fluorescence Attachment adapted Olympus BX50 microscope and a 40× objective. Five randomly selected areas from each group were photographed at 200× magnification.

### 4.5. Western Blot

SH-SY5Y cells were seeded in 75 cm^2^ flasks. After 24 h, control, mon, rap, and mon+rap groups were formed and incubated for 72 h. on ice, protein isolation was performed using RIPA lysis buffer (containing protease and phosphatase inhibitors). Concentrations of proteins were measured using a BCA kit (ABP Biosciences, Rockville, MD, USA). Proteins were separated using 12% SDS/PAGE (20 µg) and transferred to PVDF membrane (Merck Millipore, Darmstadt, Germany). The PVDF membrane was blocked with 5% skim milk powder and incubated with primary antibodies overnight at +4 °C. After 24 h of incubation, anti-rabbit horseradish peroxidase-conjugated secondary antibodies (1:20,000; BTLab) were incubated for 1 h at room temperature. Proteins were found with a WesternBright ECL HRP substrate kit (Advansta, San Jose, CA, USA) and visualized using a digital scanner (C-Digit Blot Scanner; Licor Biosciences, Lincoln, NE, USA). The primary antibodies used and their dilutions were as follows: PI3K (1:1000, cat. no. BT-PHS00765, BTLab), p-PI3K (1:1000, cat. no. BT-PHS00765, BTLab), AKT (1:1000, cat. no. BT-AP00334, BTLab), and p-AKT (1:1000, cat. no. BT-PHS00006, BTLab).

### 4.6. RT-PCR

SH-SY5Y cells were seeded in 6-well plates at 3 × 10^5^ cells/well density and incubated at 37 °C for 24 h. Control, mon, rap, and mon + rap doses were applied and incubated for 72 h in the follow-up. Total RNA was isolated according to the TRIzol reagent protocol. cDNA synthesis was performed with a Transcriptor High Fidelity cDNA Synthesis Kit according to the manufacturer’s instructions.

The mRNA-level expressions of the genes involved in the AKT pathway from the obtained cDNA were analyzed using a real-time PCR device (Applied-Biosystems, Waltham, MA, USA) according to the SYBR green method. Gene expressions were normalized using the beta-actin host gene. Fold changes of gene expressions were calculated, and experiments were repeated three times. The RT-PCR assay was performed using gene-specific validated primers. Normalization was realized using housekeeping gene β-actin (ACTB), and fold-changes were calculated by comparison with the untreated control group sample using the 2^−ΔΔCT^ method in the GeneGlobe RT² Profiler ™ PCR Array Data Analysis platform [35].

### 4.7. Statistical Analysis

Comparison of statistical significance between control and dose groups was carried out using Tukey’s or Tamhane’s method and one-way ANOVA follow-up. SPSS 23.0 statistical analysis program was used for statistical analysis evaluation, and *p* ˂ 0.05 was accepted as significant.

## 5. Conclusions

Because the nature of cancer is complex, achieving the most effective therapy with a single compound is impossible. Our data provide a solid reason to combine monensin and rapamycin in treating neuroblastoma.

In our study, we found that single and combined applications of mon and rap significantly reduced SH-SY5Y cell proliferation at low concentrations, and combining mon with rap at 72 h showed a strong synergistic effect in SH-SY5Y cells. We reported that combining mon and rap significantly reduced the mRNA expressions of the AKT, MAPK1, and p21RAS genes. We found that PI3K/AKT expression was strongly suppressed in single and combined administrations of monensin and rapamycin. In this study, we showed for the first time that monensin regulates SH-SY5Y cell proliferation by targeting the PI3K/AKT pathway and has a synergistic effect with rapamycin. Our results showed that mon alone or in combination with rap can stand as a therapeutic agent used to treat neuroblastoma. However, these data need to be supported by further preclinical studies in animal models before we can use the mon and rap combination therapy strategy in neuroblastoma patients clinically.

## Figures and Tables

**Table 1:** The toxic effects of the combination of mon (monensin) and rap (rapamycin) on SH-SY5Y cells were evaluated using the Chou Talalay combination index (CI) at 48 and 72 h.

48 h			72 h	
Mon	4 µM	8 µM	4 µM	8 µM
**50 nM rap**	0.82	1.14	0.28	0.57
**100 nM rap**	0.19	1.45	0.29	0.59
**200 nM rap**	3.17	4.09	0.28	0.59
**400 nM rap**	5.09	3.41	0.29	0.59
**800 nM rap**	8.32	5.31	0.28	0.59

CI: combination index; CI < 1: synergistic effect; CI: 1 additive; CI > 1: antagonist effect.

**Table 2:** mRNA expression changes of PI3K/AKT-pathway-related genes in mon, rap, and mon + rap treated SH-SY5Y cells compared to the control group (*p* < 0.05).

	Mon		Rap		Mon + Rap	
Gene	Fold Regulation	*p*-Value	Fold Regulation	*p*-Value	Fold Regulation	*p*-Value
***AKT***	1.08	0.555	−1.25	0.109	−1.61	0.007
***MAPK1***	−1.34	0.178	−1.19	0.977	−1.55	0.049
***P21RAS***	−1.31	0.025	−2.77	0.0007	−1.53	0.01
***PIK3C3***	−1.09	0.656	2.86	0.388	−8.43	0.258
***PTEN***	−1.52	0.585	−7.40	0.211	−20.07	0.203
***Beta-Actin***	1		1		1	

## Data Availability

The data supporting this study’s findings are available on request from the corresponding author.

## References

[1] Chung C., Boterberg T., Lucas J., Panoff J., Valteau-Couanet D., Hero B., Bagatell R., Hill-Kayser C.E. (2021). Neuroblastoma. Pediatr. Blood Cancer.

[2] Lundberg K.I., Treis D., Johnsen J.I. (2022). Neuroblastoma Heterogeneity, Plasticity, and Emerging Therapies. Curr. Oncol. Rep..

[3] Maris J.M. (2010). Recent Advances in Neuroblastoma. N. Engl. J. Med..

[4] Alexander F. (2000). Neuroblastoma. Urol. Clin. North Am..

[5] Ishola T.A., Chung D.H. (2007). Neuroblastoma. Surg. Oncol..

[6] Rajendran V., Ilamathi H.S., Dutt S., Lakshminarayana T.S., Ghosh P.C. (2018). Chemotherapeutic Potential of Monensin as an Anti-Microbial Agent. Curr. Top. Med. Chem..

[7] Zeng C., Long M., Lu Y. (2022). Monensin Synergizes with Chemotherapy in Uveal Melanoma through Suppressing RhoA. Immunopharmacol. Immunotoxicol..

[8] Yao S., Wang W., Zhou B., Cui X., Yang H., Zhang S. (2021). Monensin Suppresses Cell Proliferation and Invasion in Ovarian Cancer by Enhancing MEK1 SUMOylation. Exp. Ther. Med..

[9] Kim S.-H., Kim K.-Y., Yu S.-N., Park S.-G., Yu H.-S., Seo Y.-K., Ahn S.-C. (2016). Monensin Induces PC-3 Prostate Cancer Cell Apoptosis via ROS Production and Ca2+ Homeostasis Disruption. Anticancer. Res..

[10] Choi H.S., Jeong E.H., Lee T.G., Kim S.Y., Kim H.R., Kim C.H. (2013). Autophagy Inhibition with Monensin Enhances Cell Cycle Arrest and Apoptosis Induced by MTOR or Epidermal Growth Factor Receptor Inhibitors in Lung Cancer Cells. Tuberc. Respir. Dis..

[11] Wang X., Wu X., Zhang Z., Ma C., Wu T., Tang S., Zeng Z., Huang S., Gong C., Yuan C. (2018). Monensin Inhibits Cell Proliferation and Tumor Growth of Chemo-Resistant Pancreatic Cancer Cells by Targeting the EGFR Signaling Pathway. Sci-Entific Rep..

[12] Gu J., Huang L., Zhang Y. (2020). Monensin Inhibits Proliferation, Migration, and Promotes Apoptosis of Breast Cancer Cells via Downregulating UBA2. Drug Dev. Res..

[13] Deng Y., Zhang J., Wang Z., Yan Z., Qiao M., Ye J., Wei Q., Wang J., Wang X., Zhao L. (2015). Antibiotic Monensin Synergizes with EGFR Inhibitors and Oxaliplatin to Suppress the Proliferation of Human Ovarian Cancer Cells. Sci. Rep..

[14] Markowska A., Kaysiewicz J., Markowska J., Huczyński A. (2019). Doxycycline, Salinomycin, Monensin and Ivermectin Repositioned as Cancer Drugs. Bioorganic Med. Chem. Lett..

[15] Zhang X., Liu C., Cao Y., Liu L., Sun F., Hou L. RRS1 Knockdown Inhibits the Proliferation of Neuroblastoma Cell via PI3K/Akt/NF-ΚB Pathway. Pediatr. Res..

[16] Sartelet H., Oligny L.L., Vassal G. (2008). AKT Pathway in Neuroblastoma and Its Therapeutic Implication. Expert Rev. Anti-Cancer Ther..

[17] Opel D., Poremba C., Simon T., Debatin K.M., Fulda S. (2007). Activation of Akt Predicts Poor Outcome in Neuroblastoma. Cancer Res..

[18] Li J., Kim S.G., Blenis J. (2014). Rapamycin: One Drug, Many Effects. Cell Metab..

[19] Blagosklonny M.V. (2017). From Rapalogs to Anti-Aging Formula. Oncotarget.

[20] Waldner M., Fantus D., Solari M., Thomson A.W. (2016). New Perspectives on MTOR Inhibitors (Rapamycin, Rapalogs and TORKinibs) in Transplantation. Br. J. Clin. Pharmacol..

[21] Chen X.G., Liu F., Song X.F., Wang Z.H., Dong Z.Q., Hu Z.Q., Lan R.Z., Guan W., Zhou T.G., Xu X.M. (2010). Rapamy-cin Regulates Akt and ERK Phosphorylation through MTORC1 and MTORC2 Signaling Pathways. Mol. Carcinog..

[22] King D., Yeomanson D., Bryant H.E. (2015). PI3King the Lock: Targeting the PI3K/Akt/MTOR Pathway as a Novel Therapeutic Strategy in Neuroblastoma. J. Pediatr. Hematol./Oncol..

[23] Verma S.P., Das P. (2018). Monensin Induces Cell Death by Autophagy and Inhibits Matrix Metalloproteinase 7 (MMP7) in UOK146 Renal Cell Carcinoma Cell Line. Vitr. Cell. Dev. Biol. Anim..

[24] Knight Z.A., Shokat K.M. (2007). Chemically Targeting the PI3K Family. Biochem. Soc. Trans..

[25] Liu P., Cheng H., Roberts T.M., Zhao J.J. (2009). Targeting the Phosphoinositide 3-Kinase Pathway in Cancer. Nat. Rev. Drug Discov..

[26] Wong K.-K., Engelman J.A., Cantley L.C. (2010). Targeting the PI3K Signaling Pathway in Cancer. Curr. Opin. Genet. Dev..

[27] Lin X., Han L., Weng J., Wang K., Chen T. (2018). Rapamycin Inhibits Proliferation and Induces Autophagy in Human Neuro-blastoma Cells. Biosci. Rep..

[28] Johnsen J.I., Segerström L., Orrego A., Elfman L., Henriksson M., Kågedal B., Eksborg S., Sveinbjörnsson B., Kogner P. (2008). Inhibitors of Mammalian Target of Rapamycin Downregulate MYCN Protein Expression and Inhibit Neuroblastoma Growth in Vitro and in Vivo. Oncogene.

[29] Moreno-Smith M., Lakoma A., Chen Z., Tao L., Scorsone K.A., Schild L., Aviles-Padilla K., Nikzad R., Zhang Y., Chakraborty R. (2017). P53 Nongenotoxic Activation and MTORC1 Inhibition Lead to Effective Combination for Neuroblas-toma Therapy. Clin. Cancer Res. Off. J. Am. Assoc. Cancer Res..

[30] Zhang L., Smith K.M., Chong A.L., Stempak D., Yeger H., Marrano P., Thorner P.S., Irwin M.S., Kaplan D.R., Baruchel S. (2009). In Vivo Antitumor and Antimetastatic Activity of Sunitinib in Preclinical Neuroblastoma Mouse Model. Neoplasia.

[31] Liao J., Jiang L., Wang C., Zhao D., He W., Zhou K., Liang Y. (2022). FoxM1 Regulates Proliferation and Apoptosis of Human Neuroblastoma Cell through PI3K/AKT Pathway. Fetal Pediatr. Pathol..

[32] Fresno Vara J.Á., Casado E., de Castro J., Cejas P., Belda-Iniesta C., González-Barón M. (2004). PI3K/Akt Signalling Pathway and Cancer. Cancer Treat. Rev..

[33] Noorolyai S., Shajari N., Baghbani E., Sadreddini S., Baradaran B. (2019). The Relation between PI3K/AKT Signalling Pathway and Cancer. Gene.

[34] Fulda S. (2009). The PI3K/Akt/MTOR Pathway as Therapeutic Target in Neuroblastoma. Curr. Cancer Drug Targets.

[35] Kocoglu S.S., Seçme M., Elmas L. (2022). Erianin, a Promising Agent in the Treatment of Glioblastoma Multiforme Triggers Apoptosis in U373 and A172 Glioblastoma Cells. Arch. Biol. Sci..

[36] Tezcan B., Serter S., Kiter E., Tufan A.C. (2010). Dose Dependent Effect of C-Type Natriuretic Peptide Signaling in Glycosaminoglycan Synthesis during TGF-Β1 Induced Chondrogenic Differentiation of Mesenchymal Stem Cells. J. Mol. Histol..

